# A multi-dimensional omics framework identifies GPR35 as a driver of M2 macrophage activation and poor prognosis in colorectal cancer

**DOI:** 10.3389/fimmu.2026.1783260

**Published:** 2026-02-18

**Authors:** Shen Guan, Liangchen Zhu, Yue Tian, Hong Chen, Yiqing Jiang, Chenshen Huang, Yuanying Shi, Dajia Lin

**Affiliations:** 1 Department of Colorectal Surgery, Clinical Oncology School of Fujian Medical University, Fujian Cancer Hospital, Fuzhou, China; 2 Shanghai Tongji Hospital, School of Medicine, Tongji University, Shanghai, China; 3 Department of Cardiovascular Surgery, Fujian Medical University Union Hospital, Fuzhou, Fujian, China; 4 Fuzhou University Affiliated Provincial Hospital, School of Medicine, Fuzhou University, Fuzhou, China; 5 School of Mathematical Sciences, Tongji University, Shanghai, China; 6 Shengli Clinical Medical College of Fujian Medical University, Fujian Provincial Hospital, Fuzhou, China

**Keywords:** GPR35, machine learning (ML), multi-omcis, NMF (nonnegative matrix factorization), tumor microenveronment (TME)

## Abstract

**Introduction:**

Colorectal cancer (CRC) remains a leading cause of global cancer mortality, with therapeutic outcomes heavily reliant on the tumor microenvironment (TME). While immunotherapy has revolutionized treatment for distinct subsets, the mechanisms driving immune evasion in the majority of patients remain elusive.

**Methods:**

In this study, we constructed a comprehensive single-cell atlas of the CRC TME by integrating multi-cohort scRNA-seq data.

**Results:**

Through non-negative matrix factorization (NMF), we identified nine intratumoral heterogeneity meta-programs (MPs), among which MP8 was robustly linked to M2 macrophage activation. High-dimensional WGCNA further pinpointed GPR35 as a master regulator within the MP8-associated gene network. Clinical analysis across four independent cohorts validated GPR35 as a significant predictor of poor prognosis. Functionally, GPR35 knockdown *in vitro* markedly impaired CRC cell proliferation, migration, and invasion. Mechanistically, high GPR35 expression orchestrated an immune-excluded microenvironment characterized by diminished cytotoxic T cell and NK cell recruitment, yet paradoxically elevated immune checkpoint expression. Furthermore, GPR35 expression was negatively correlated with eight established immunotherapy response signatures and associated with aggressive mutational landscapes.

**Discussion:**

Collectively, our findings identify GPR35 as a novel cancer cell-intrinsic driver of immune evasion and immunotherapy resistance, positioning it as a promising therapeutic target to sensitize "cold" CRC tumors to immune checkpoint blockade.

## Introduction

Colorectal cancer (CRC) remains one of the most prevalent and deadly malignancies worldwide, accounting for approximately 10% of all cancer cases and deaths globally. Its high mortality is largely attributed to late-stage diagnosis, metastatic progression, and therapeutic resistance (1). CRC is a molecularly and clinically heterogeneous disease, characterized by distinct genetic, epigenetic, and microenvironmental features that influence disease behavior and treatment outcomes (2, 3). In recent years, immunotherapy, particularly immune checkpoint blockade (ICB), has revolutionized oncology by offering durable responses in several cancer types. However, in CRC, its efficacy is largely restricted to the minority of patients with microsatellite instability-high (MSI-H) or mismatch repair-deficient (dMMR) tumors (4). The majority of patients with microsatellite-stable (MSS) CRC exhibit primary resistance to immunotherapy, underscoring the urgent need to elucidate the mechanisms of immune evasion and identify novel targets to sensitize these tumors to immune-mediated attack. Emerging evidence suggests that GPR35 acts as a critical link between cancer cell-intrinsic signaling and the immune microenvironment, yet its specific role in driving immune evasion in CRC remains to be fully elucidated.

The tumor microenvironment (TME) is a dynamic and complex ecosystem composed of malignant cells, immune infiltrates, cancer-associated fibroblasts, endothelial cells, and extracellular matrix components (5, 6). This intricate network mediates critical processes, including immune surveillance, tumor progression, and therapeutic response (7, 8). The development of single-cell transcriptomic technologies has provided unprecedented resolution for dissecting cellular heterogeneity, identifying rare cell states, and mapping intercellular communication within the TME (9). One key cellular component that bridges tumor microenvironment (TME) remodeling and anti-tumor immunity is the M2 macrophage (10). M2 macrophages are immunosuppressive cells that secrete anti-inflammatory cytokines such as IL-10 and TGF-β, promote angiogenesis and matrix remodeling, and inhibit T-cell function. Tumors with abundant M2 macrophage infiltration are generally more resistant to immunotherapy; however, reprogramming M2 macrophages toward an immunostimulatory phenotype may help overcome immune escape (11). Therefore, identifying key regulators of M2 macrophage polarization within cancer cells could unveil new strategies to reprogram the TME toward an immunogenic state.

G protein-coupled receptor 35 (GPR35) is an orphan receptor with emerging roles in inflammation, metabolism, and cancer (12). It is expressed in various immune cells and epithelial tissues. In cancer, GPR35 has been implicated in cell proliferation, migration, and metastatic potential in certain contexts, yet its precise function and mechanistic role in CRC, particularly in sculpting the immunosuppressive TME, remain largely unexplored. Although its expression and function have been studied in immune cells and certain epithelial contexts, its role in CRC, particularly in shaping the immune landscape, remains poorly understood. Preliminary evidence suggests that GPR35 may influence intracellular signaling pathways related to cell survival, migration, and immune modulation, but systematic studies linking GPR35 to CRC progression and immunotherapy resistance are lacking. Given its signaling potential and expression pattern, we hypothesize that GPR35 may serve as a critical nexus linking cancer cell intrinsic programs to extrinsic immune modulation.

In this study, we hypothesize that cancer cell-intrinsic GPR35 expression modulates the TME and contributes to immunotherapy resistance in CRC. By integrating bulk transcriptomics from multiple cohorts, scRNA-seq data, and *in vitro* functional validation, we aim to systematically dissect the CRC TME (13). Specifically, we first comprehensively map the cellular and transcriptional landscape at single-cell resolution to identify meta-programs driving intratumoral heterogeneity and M2 macrophage activation. Building on this high-resolution map, we aim to screen and validate key regulator genes associated with patient prognosis. Ultimately, this study focuses on characterizing the immunological and therapeutic implications of the top candidate, GPR35, in driving CRC progression and facilitating immune evasion.

## Materials and methods

### Data acquisition and preprocessing

Transcriptomic data and corresponding clinical information from CRC patients were obtained from five publicly available cohorts, including TCGA-CRC (14), GSE103479 (15), GSE72968 (16), GSE41258 (17), and GSE72970 (16). We obtained scRNA-seq data from CRC samples of AHCA (5738 cells), GutCellAtlasColon (25104 cells), GutCellAtlasIntestine (41863 cells), TabulaSapiens (8228 cells), and TissueImmuneCellAtlas (453 cells). The R package Seurat was used to process the scRNA-seq data (data normalization, scaling, and integration) (18, 19). Quality control was performed before integration. Cells with unique feature counts (nFeature_RNA) less than 200 or greater than 6000, and cells where >20% of counts originated from mitochondrial genes were filtered out to remove low-quality cells and potential doublets. To correct for batch effects arising from different datasets and sequencing platforms, we applied the Harmony algorithm. The integration performance was assessed visually using UMAP plots to ensure the intermingling of cells from different cohorts while preserving biological clusters. The M2 macrophage signature gene list was obtained from the previous study (20, 21).

### Computational analysis

The R package InferCNV was used to identify cancer cells among the TME cells. UMAP was used for two-dimensional visualization (22). A reference set of normal epithelial cells from healthy colon samples was used to infer copy number variation. Cells with elevated large-scale chromosomal aberrations were annotated as cancer cells. To dissect transcriptional heterogeneity within the malignant compartment, non-negative matrix factorization (NMF) was performed on the cancer cell expression matrix. The optimal rank (k = 9) was determined based on cophenetic correlation and the residual sum of squares. The meta-programs (MPs) highlighting intratumor heterogeneity were identified as previously described via the R package GeneNMF (23). Gene modules associated with MP8 were identified via the R package hdWGCNA (24). A soft-thresholding power of six was selected to achieve a scale-free topology fit. The connections between GPR35 and the stromal score, immune score, ESTIMATE score, and tumor purity were determined via the ESTIMATE algorithm (25). The association between GPR35 and nine established immunotherapy response signatures, CYT (26), IFNγ (27), Ayers expIS (27), T-cell-inflamed (27), Roh IS (28), Davoli IS (29), Chemokines (30), RIR (31), and ICBnetIS (6) was explored. The Metascape platform and GSEA of GO terms were used for functional annotation of GPR35 (32). The association between GPR35 and immune cells was predicted through the MCPcounter (33), Porpimol’s study via ssGSEA (34), and TIMER (29). The cancer immune cycles related to GPR35 were quantified (6), with step-specific gene signatures scored via ssGSEA. Somatic mutation landscapes related to GPR35 were visualized and compared using maftools (35).

### *In vitro* validation

Human CRC cell lines, including LOVO and RKO, were maintained in DMEM containing 10% FBS and 1% penicillin/streptomycin under standard culture conditions at 37 °C in a 5% CO_2_ humidified atmosphere. To investigate the functional role of GPR35, small interfering RNA (siRNA) targeting GPR35 and a non-targeting control (NC) siRNA were transfected into cells according to the manufacturer’s protocol. Cell proliferation capacity was assessed using the EdU incorporation assay. Migratory and invasive capacities were evaluated using Transwell chambers without or with Matrigel coating, respectively. After 48 hours of incubation, cells that migrated/invaded through the membrane were stained and counted.

### Statistical analysis

For comparisons between two groups with non-normal distribution, the Wilcoxon rank-sum test was used. For survival analysis, the log-rank test was employed. P-values from multiple comparisons were adjusted using the Benjamini-Hochberg False Discovery Rate (FDR) method. An adjusted p-value (FDR) < 0.05 was considered statistically significant.

## Results

### Single-cell deconvolution defines the CRC tumor microenvironment

**Figure 1:**
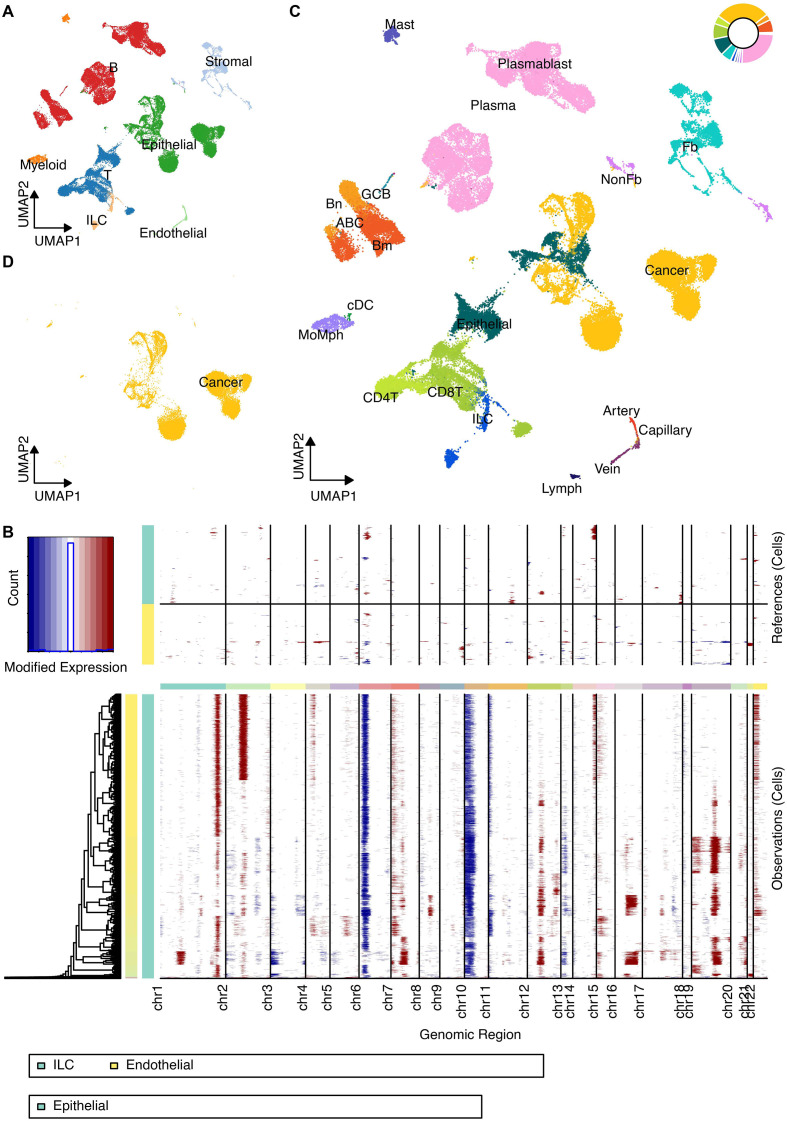
Definition of TME cells at the scRNA-seq level. **(A)** UMAP projection of all integrated cells, color-coded by major cell types (B cells, T cells, Epithelial cells, Stromal cells, Endothelial cells, Innate Lymphoid Cells, and Myeloid cells). **(B)** Heatmap displaying the inferred Copy Number Variation (CNV) scores across TME cells to distinguish malignant from non-malignant cells. **(C)** Detailed UMAP clustering of the non-malignant TME compartment showing minor cell subsets. **(D)** UMAP highlighting the identified malignant epithelial cells based on CNV analysis.

**Figure 2:**
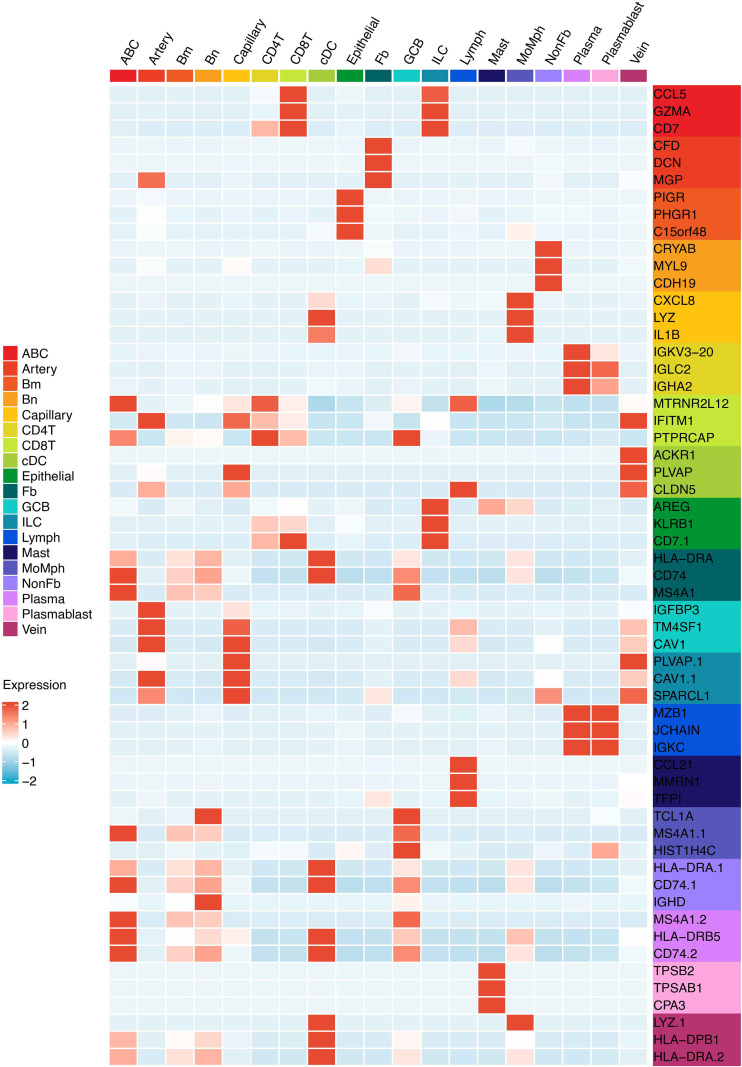
The heatmap shows the marker genes of minor TME cell types.

Unified analysis of scRNA-seq data from multiple public cohorts resolved the major cellular compartments within the CRC TME, including B cells, T cells, Epithelial cells, Stromal cells, Endothelial cells, Innate Lymphoid Cells (ILC), and Myeloid cells (**Figure 1A**). InferCNV analysis distinguished malignant epithelial cells from the surrounding stroma (**Figures 1B, D**), while further clustering revealed distinct minor TME subsets, validated by specific marker gene expression (**Figure 1C**, **2**).

### NMF identifies intratumoral heterogeneity programs linking MP8 to M2 macrophage activation

**Figure 3:**
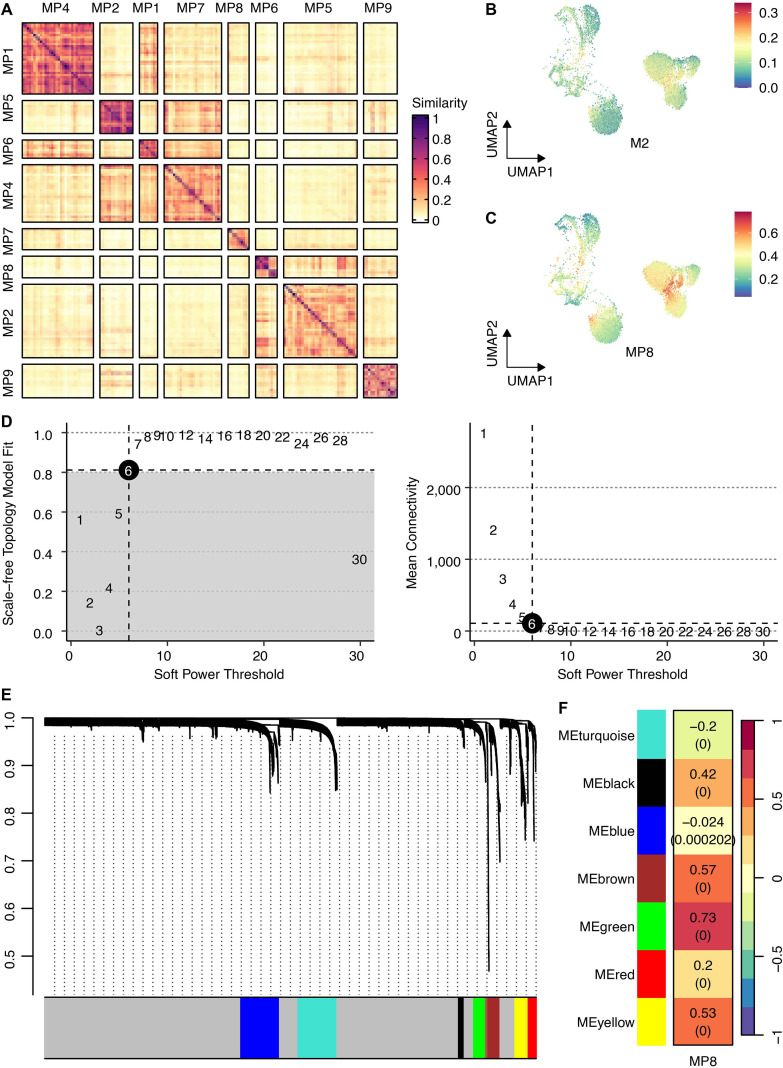
Identification of intratumoral heterogeneity meta-programs and the M2 macrophage-associated gene regulatory network. **(A)** Heatmap shows Jaccard similarity indices for comparisons among nine robust NMF programs in cancer cells. The programs are ordered by clustering and grouped into MPs. **(B)** UMAP shows the M2 scores across cancer cells. **(C)** UMAP shows the MP8 signature scores across cancer cells. **(D)** The connection between scale-free topology model fit, mean connectivity, and soft power threshold. **(E)** Waterfall plot shows the distribution of gene modules. **(F)** The correlation between the MP8 signature and gene modules.

Non-negative matrix factorization of cancer cell transcriptomes revealed nine robust MPs representing core biological processes (**Figure 3A**). Among these, MP8 demonstrated the strongest association with a validated M2 macrophage gene signature (**Figures 3B, C**), suggesting its role in regulating a potential immunogenic state within tumor cells.

### hdWGCNA identifies the MP8-associated green module and nominates GPR35 as a key regulator

**Figure 4:**
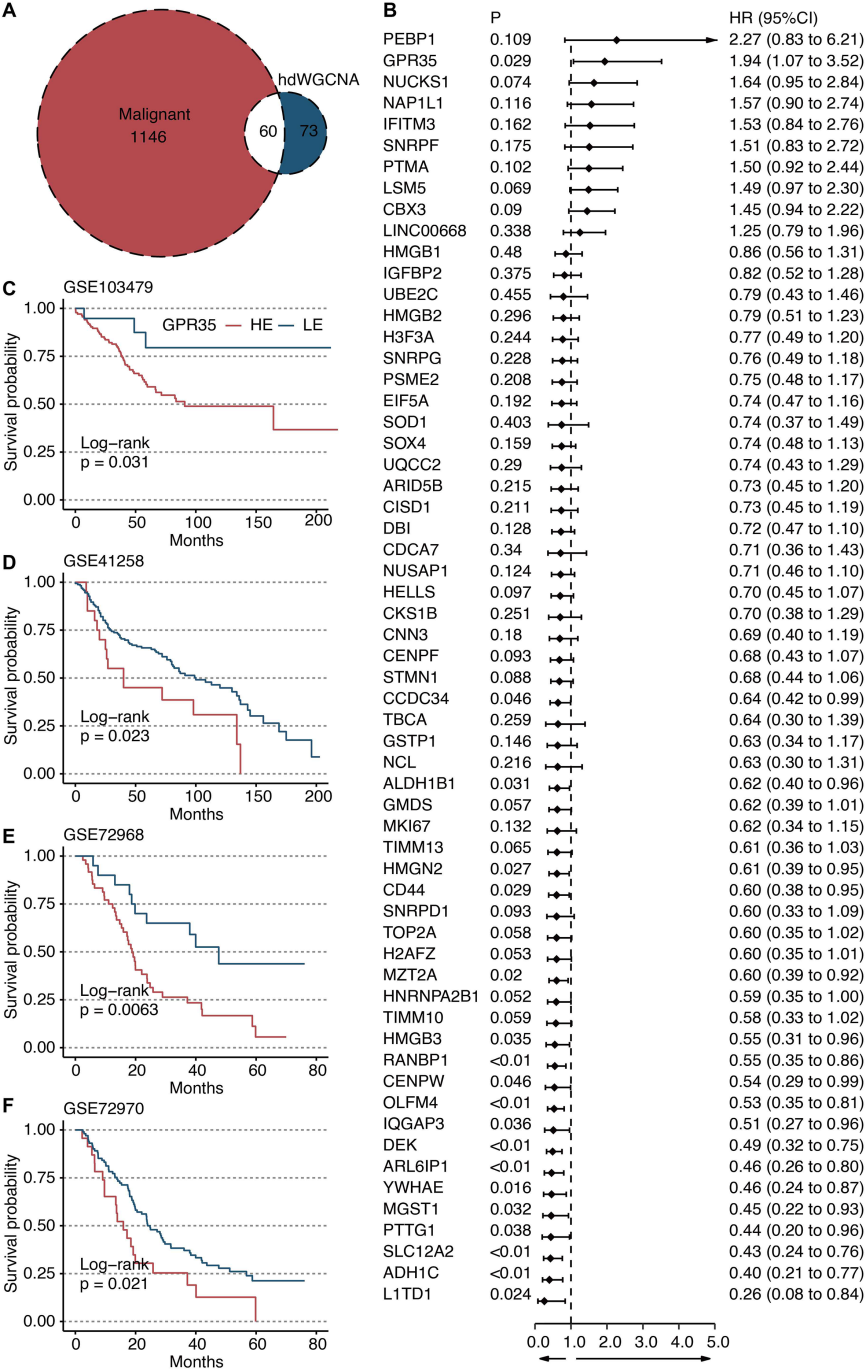
Identification of GPR35 as a hazardous gene. **(A)** Venn plot shows the intersecting genes between green module-derived genes and genes upregulated in cancer cells compared with other TME cells. **(B)** Univariate Cox regression analysis on 60 intersecting genes. Survival curves of GPR35-based groups in **(C)** GSE103479, **(D)** GSE41258, **(E)** GSE72968, and **(F)** GSE72970 datasets.

To elucidate the gene network underlying MP8, we performed hdWGCNA. Network construction employed a soft power threshold of 6, optimized for scale-free topology (**Figure 3D**). This yielded multiple co-expression modules (**Figure 3E**), among which the green module showed the highest positive correlation with the MP8 signature (r = 0.73) (**Figure 3F**). This module contained 133 genes. Intersection of these genes with 1,206 transcripts significantly upregulated in cancer cells versus all other TME cells yielded 60 overlapping candidates (**Figure 4A**). Univariate Cox regression analysis prioritized GPR35 as the most significant prognostic hazard gene from this intersection (**Figure 4B**).

### GPR35 expression is a robust prognostic marker across independent CRC cohorts

Kaplan-Meier survival analysis consistently demonstrated that high tumor expression of GPR35 was associated with significantly shorter overall survival across four independent validation cohorts: GSE103479 (**Figure 4C**), GSE41258 (**Figure 4D**), GSE72968 (**Figure 4E**), and GSE72970 (**Figure 4F**). This establishes GPR35 as a reproducible biomarker of poor prognosis in CRC. To further validate the prognostic value of GPR35, we performed multivariate Cox regression analysis adjusting for age, gender, and tumor stage. GPR35 remained an independent prognostic factor (HR > 1, P < 0.05), indicating its robustness beyond standard clinical variables.

### *In vitro* functional validation confirms the pro-tumorigenic role of GPR35

**Figure 5:**
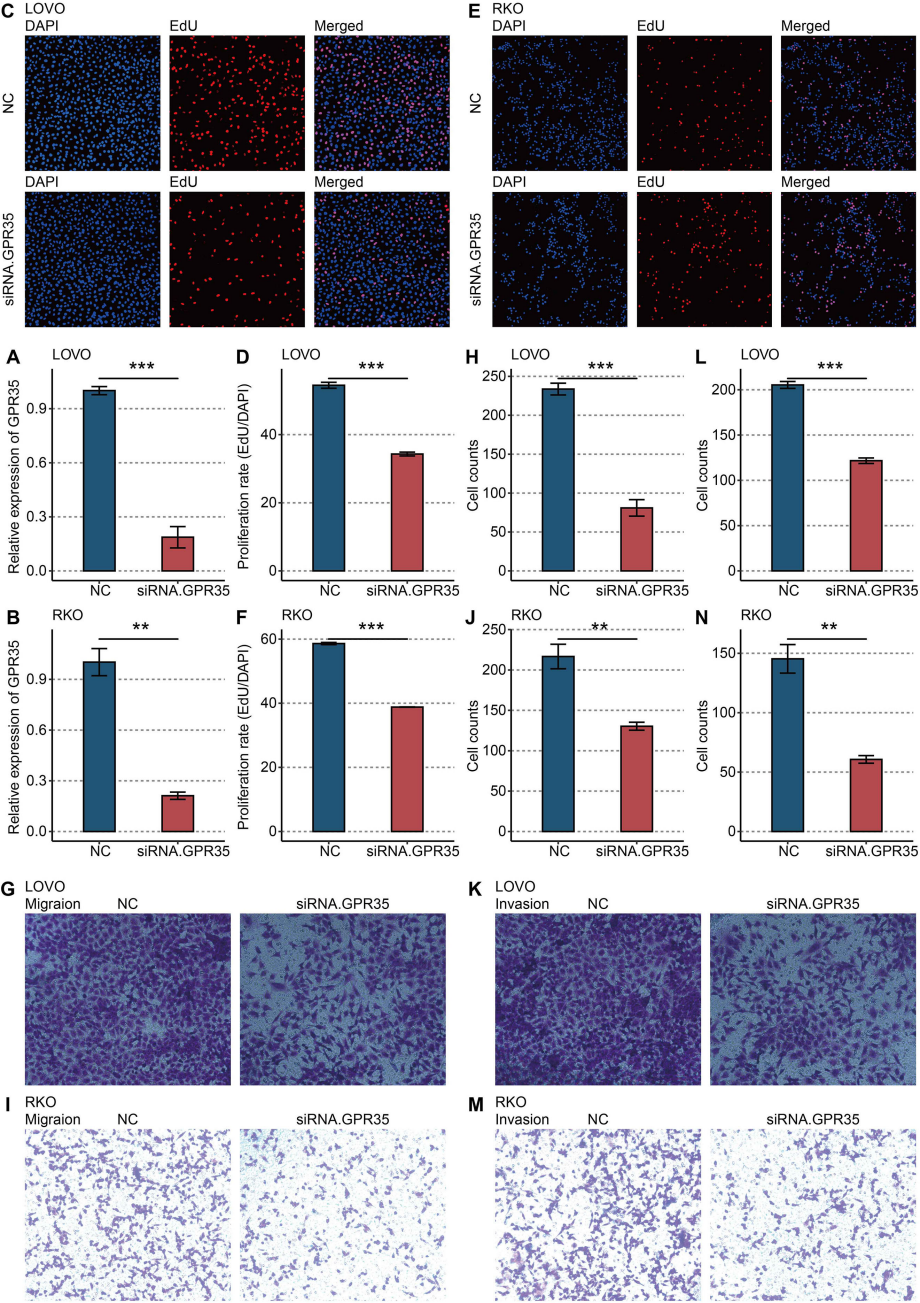
*In vitro* validation on GPR35. **(A)** RT-qPCR shows the knockdown efficacy of siRNA in LOVO cells. **(B)** RT-qPCR shows the knockdown efficacy of siRNA in RKO cells. **(C)** Representative images of EdU assay in NC and siRNA-GPR35 groups in LOVO cells. **(D)** Statistical analysis of EdU assay in LOVO cells. **(E)** Representative images of EdU assay in NC and siRNA-GPR35 groups in RKO cells. **(F)** Statistical analysis of EdU assay in RKO cells. **(G)** Representative images of the Transwell assay for migration detection in the NC and siRNA-GPR35 groups in LOVO cells. **(H)** Statistical analysis of the Transwell assay for migration detection in LOVO cells. **(I)** Representative images of the Transwell assay for migration detection in the NC and siRNA-GPR35 groups in RKO cells. **(J)** Statistical analysis of the Transwell assay for migration detection in RKO cells. **(K)** Representative images of the Transwell assay for invasion detection in NC and siRNA-GPR35 groups in LOVO cells. **(L)** Statistical analysis of the Transwell assay for invasion detection in LOVO cells. **(M)** Representative images of the Transwell assay for invasion detection in NC and siRNA-GPR35 groups in RKO cells. **(N)** Statistical analysis of the Transwell assay for invasion detection in RKO cells. **, P < 0.01; ***, P < 0.001.

siRNA-mediated knockdown of GPR35 in LOVO and RKO CRC cell lines was confirmed at the mRNA level (**Figures 5A, B**). Functional assays revealed that GPR35 depletion significantly impaired cellular proliferation, as measured by EdU incorporation (**Figures 5C–F**). Furthermore, GPR35 knockdown markedly reduced the migratory (**Figures 5G–J**) and invasive (**Figures 5K–N**) capacities of both cell lines in Transwell assays, confirming its direct role in promoting aggressive CRC phenotypes.

### GPR35 is linked to an immunosuppressive transcriptional landscape

**Figure 6:**
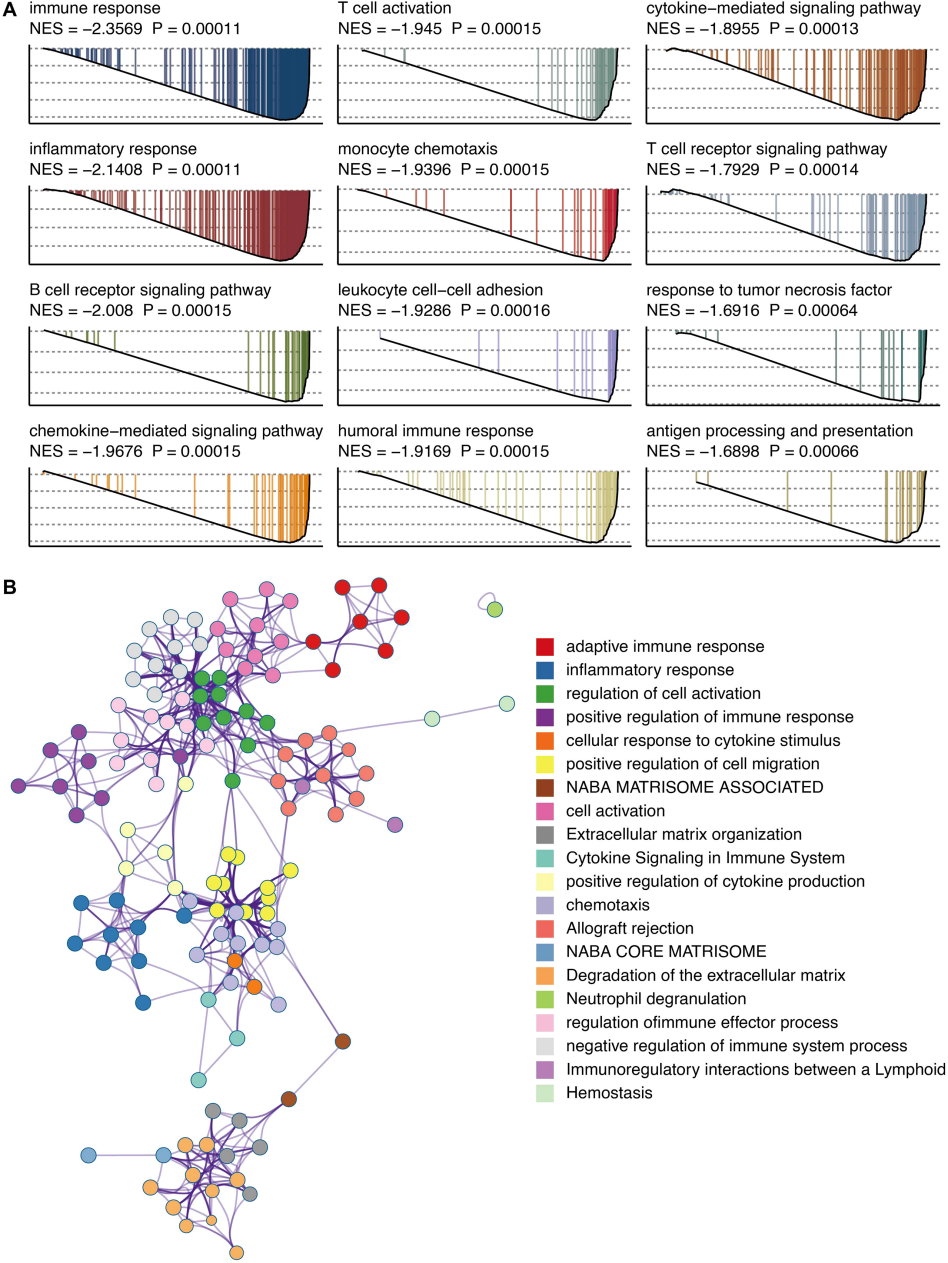
Functional annotation of GPR35. **(A)** GSEA of GO pathways related to GPR35. **(B)** Metascape-based pathways related to GPR35.

GSEA revealed that high GPR35 expression was negatively associated with key immune-activating pathways, including “Immune response,” “T cell activation,” and “T cell receptor signaling pathway” (**Figure 6A**). Metascape pathway annotation corroborated these findings, highlighting the suppression of immune system processes (**Figure 6B**).

### GPR35 expression correlates with an immune-excluded microenvironment and altered cancer-immunity cycle

**Figure 7:**
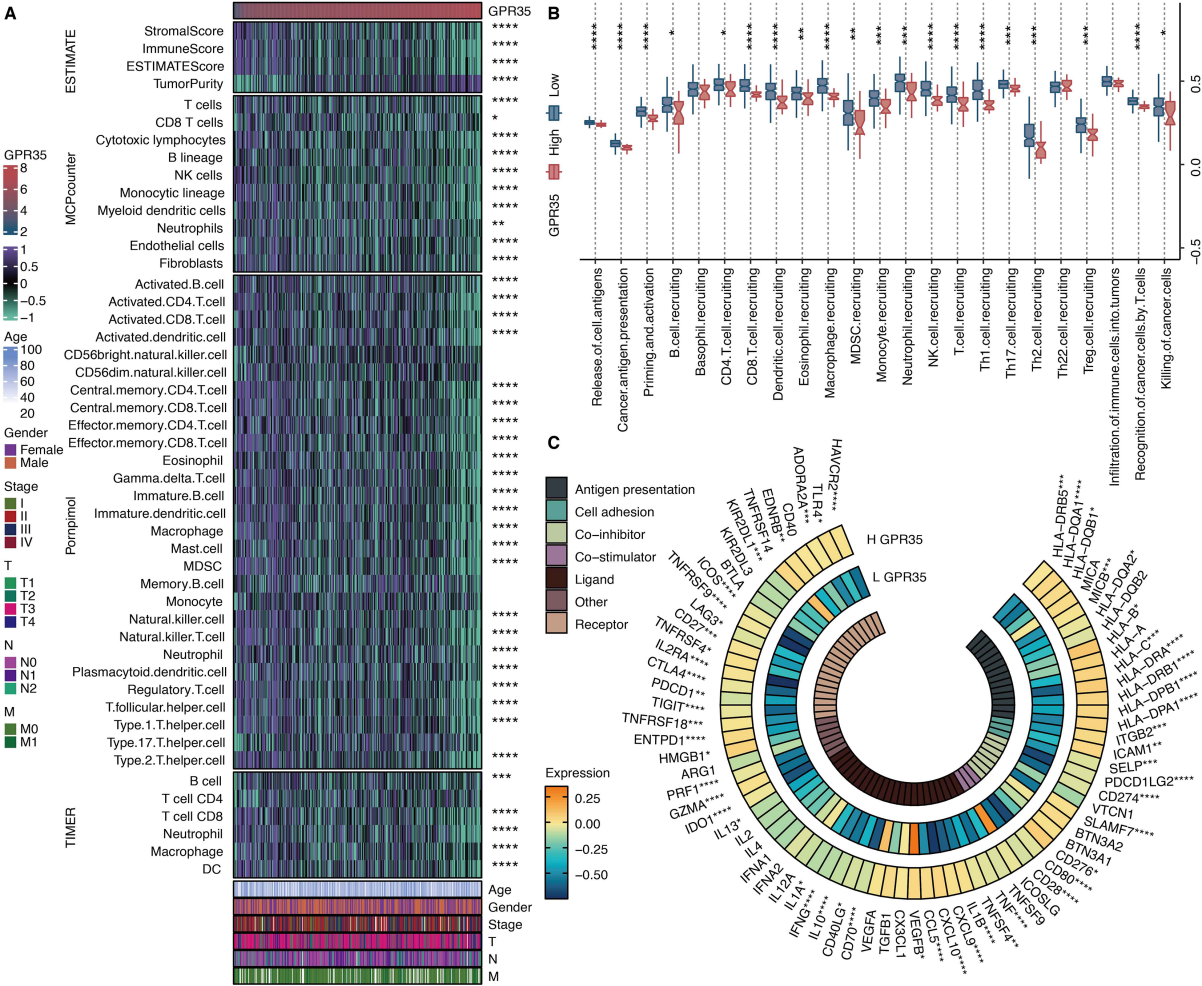
Immune features of GPR35. **(A)** Heatmap shows the correlation between GPR35 and microenvironment scores, MCPcounter-based immune cells, Pornpimol-based immune cells, and TIMER-based immune cells. **(B)** The levels of cancer immune cycles in GPR35-based groups. **(C)** The circos plot shows the correlation between GPR35 and immune modulators. *, P < 0.05; **, P < 0.01; ***, P < 0.001; ****, P < 0.0001.

Analysis of microenvironment scores revealed that high GPR35 expression was inversely correlated with stromal, immune, and ESTIMATE scores but positively correlated with tumor purity (**Figure 7A**). This suggested an immunologically “cold” tumor niche. Consistent with this, bioinformatics analyses using MCPcounter, ssGSEA, and TIMER algorithms consistently demonstrated that elevated GPR35 levels were associated with significantly reduced inferred infiltration of multiple anti-tumor immune cell types, including CD4 T cells, CD8 T cells, dendritic cells, and neutrophils (**Figure 7A**). To functionally dissect this immune exclusion, we quantified key steps of the cancer-immunity cycle. This revealed that tumors with high GPR35 expression exhibited significant defects in the recruitment of both T cells and natural killer (NK) cells (**Figure 7B**). Paradoxically, despite the overall paucity of immune infiltrates, GPR35 expression showed a significant positive correlation with the transcriptional levels of critical immune checkpoint molecules, such as PD-L1 and CTLA-4 (**Figure 7C**), indicating a co-suppressive microenvironment.

### GPR35 expression predicts diminished response to immunotherapy

**Figure 8:**
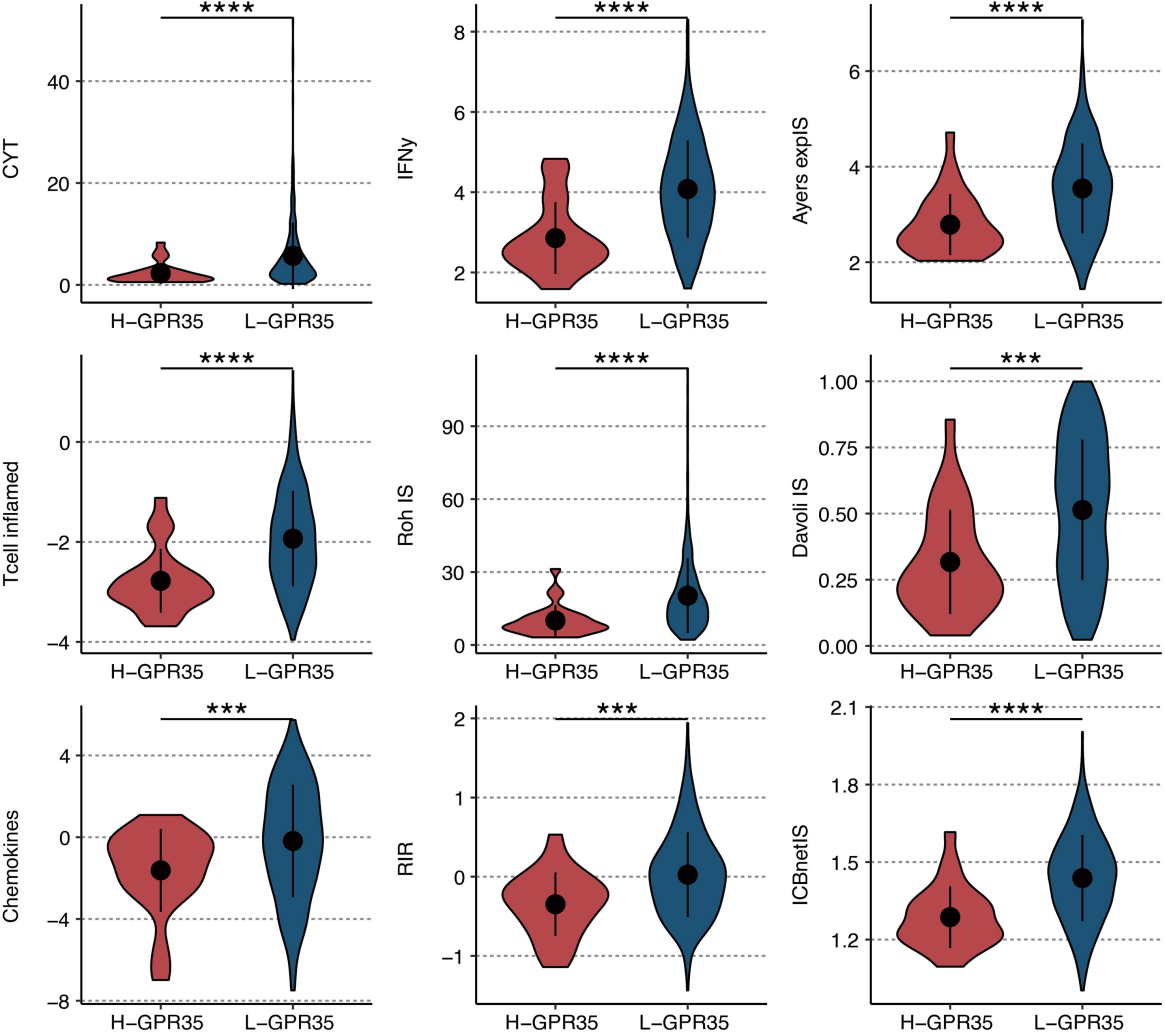
Immunotherapy response prediction of GRP35. The levels of nine immunotherapeutic signatures in GPR35-based groups. ***, P < 0.001; ****, P < 0.0001.

Evaluation using nine established transcriptional signatures predictive of immunotherapy response demonstrated that high GPR35 expression was associated with significantly lower activity scores for eight of these signatures, including CYT, IFNγ, and T-cell-inflamed (**Figure 8**). This pattern suggests that GPR35-high tumors exhibit a broadly suppressed adaptive immune microenvironment.

### Distinct somatic mutation landscapes associate with GPR35 expression

Analysis of somatic mutations revealed that tumors with high GPR35 expression were characterized by a distinct mutation profile. APC and TP53 were the most frequently mutated genes in this group (**Supplementary Figure S1A**). A comparative analysis identified several genes with significantly different mutation frequencies between the high- and low-GPR35 groups, including the epigenetic regulator KMT2D, the netrin receptor UNC5B, and the potassium channel KCNH8 (**Supplementary Figure S1B**, **S2**).

## Discussion

Our integrative multi-omics study provides a comprehensive single-cell atlas of the CRC TME and systematically identifies GPR35 as a novel cancer cell-intrinsic driver of poor prognosis and immune evasion. The robust association between high GPR35 expression and adverse survival across multiple independent cohorts solidifies its clinical relevance as a promising prognostic biomarker.

The functional validation *in vitro* unambiguously establishes the pro-oncogenic role of GPR35, demonstrating its necessity for the proliferation, migration, and invasion of CRC cells. This positions GPR35 as a potential direct therapeutic target. More critically, our bioinformatic analyses uncover a profound and consistent link between GPR35 and a profoundly immunosuppressive TME. The strong negative correlations with T cell activation pathways, cytotoxic immune cell infiltration (CD4/CD8 T cells, DCs), and essential steps in the cancer-immunity cycle (e.g., T/NK cell recruitment) collectively describe an “immune-excluded” phenotype (36). This exclusion is paradoxically accompanied by increased expression of immune checkpoint molecules, such as PD-L1, suggesting that an adaptive immune resistance mechanism may be concurrently activated, further complicating immune-mediated tumor elimination (37).

The predictive power of GPR35 for immunotherapy resistance is a particularly significant translational finding of our study. The uniformly low activity of eight key immunotherapy response signatures in GPR35-high tumors indicates that GPR35 may orchestrate a broad-spectrum resistance program, potentially independent of microsatellite status. This is crucial because most CRC cases are MSS and currently derive minimal benefit from immunotherapy. GPR35 could thus serve as a biomarker to identify MSS patients unlikely to respond to immune checkpoint inhibitors, thereby sparing them from ineffective and potentially toxic treatments. The isolated elevation of the chemokine in GPR35-high tumors is intriguing and warrants mechanistic investigation. It may represent a maladaptive or immunosuppressive chemokine milieu that fails to productively recruit cytotoxic lymphocytes, or recruits immunosuppressive myeloid populations such as MDSCs or M2-like macrophages. Future studies should profile the chemokine and cytokine secretion profiles of GPR35-overexpressing tumor cells and determine their functional impact on immune cell migration and polarization.

The distinct mutational landscape associated with high GPR35 expression provides additional context. The enrichment of canonical APC (38) and TP53 (39) mutations aligns this subtype with more aggressive, chromosomally unstable CRC pathways. The differential mutation of genes like KMT2D (40), a histone methyltransferase involved in chromatin remodeling, and UNC5B (41), a dependence receptor implicated in apoptosis and angiogenesis, points to potential cooperative genetic events that could synergize with GPR35 signaling to shape both tumor evolution and the immune contexture. For instance, loss-of-function mutations in KMT2D can lead to global alterations in histone methylation, potentially silencing tumor suppressor genes and immune-stimulatory genes, thereby reinforcing the immunosuppressive program driven by GPR35. The co-occurrence of these mutations suggests a possible convergent evolutionary pathway toward immune evasion and therapy resistance.

While our study establishes strong correlative and functional links, several key mechanistic questions remain. The precise molecular mechanism by which GPR35 signaling in cancer cells extrinsically suppresses immune cell infiltration and function is unknown. As an orphan receptor, the relevant endogenous or tumor-derived ligands for GPR35 in the CRC TME need to be identified. Candidates include kynurenic acid, a tryptophan metabolite known to activate GPR35 and accumulate in immunosuppressive environments, and 5-hydroxyindoleacetic acid (5-HIAA), a serotonin metabolite recently shown to promote neutrophil recruitment via GPR35 in inflammatory contexts (23, 36). It is plausible that tumor-derived ligands activate GPR35 in an autocrine manner, leading to the secretion of immunosuppressive factors (e.g., IL-10, TGF-β, VEGF) or the downregulation of chemokines required for T cell homing (e.g., CXCL9, CXCL10). Downstream effector pathways must also be delineated—whether GPR35 exerts its effects primarily through Gαi/o protein-mediated inhibition of cAMP, through β-arrestin-mediated scaffolding and receptor internalization, or through cross-talk with other oncogenic pathways, such as the Wnt/β-catenin pathway, which is frequently dysregulated in APC-mutant CRC.

Future work must validate these interactions *in vivo* using syngeneic or genetically engineered immunocompetent mouse models of CRC. Such models would enable the dissection of cell-type-specific functions of GPR35, whether its tumor-promoting and immunosuppressive effects are primarily driven by expression in cancer cells or by expression in stromal or immune cells (e.g., myeloid cells). Furthermore, the clinical predictive value of GPR35 should be prospectively tested in cohorts of CRC patients treated with immunotherapy. Assessing GPR35 expression (via RNA sequencing or immunohistochemistry) in pre-treatment tumor biopsies from clinical trials of anti-PD-1/PD-L1 agents would determine its utility as a companion diagnostic.

A primary limitation of this study is the lack of *in vivo* validation using immunocompetent animal models (42). Future work must employ syngeneic or genetically engineered mouse models of CRC to confirm the causal role of tumor cell-intrinsic GPR35 in shaping an immunosuppressive TME and driving immunotherapy resistance *in vivo*. Furthermore, the precise downstream signaling pathways (e.g., cAMP, β-arrestin, or cross-talk with Wnt/β-catenin) through which GPR35 exerts its pro-tumorigenic and immunomodulatory effects remain unknown. Systematic approaches such as phosphoproteomics or RNA-seq following GPR35 modulation in relevant models are needed to map these networks. Additionally, the cohorts analyzed were primarily from public databases with limited diversity in race and ethnicity. Multi-center prospective studies incorporating patient samples from varied geographic and ethnic backgrounds, as well as the use of patient-derived organoids (43, 44), are essential to establish the robustness and generalizability of GPR35 as a biomarker. Furthermore, potential selection bias exists as the cohorts analyzed were retrospective and sourced from public databases. To mitigate this, future multi-center prospective studies incorporating patient samples from varied geographic and ethnic backgrounds are essential to generalize our findings.

Our study nominates GPR35 as both a prognostic biomarker and a potential therapeutic target to overcome immunotherapy resistance in MSS CRC. A direct translational application would be to evaluate GPR35 expression in pretreatment biopsies from patients enrolled in ICB trials as a predictive biomarker. From a therapeutic perspective, developing selective GPR35 antagonists or leveraging siRNA/nanoparticle delivery systems to silence GPR35 in tumors could be promising strategies. Combining GPR35 inhibition with anti-PD-1/PD-L1 therapy might synergistically reverse the immune-excluded phenotype. This dual-target approach could potentially convert “cold” tumors into “hot” ones, thereby expanding the reach of immunotherapy in colorectal cancer.

## Data Availability

The original contributions presented in the study are included in the article/**Supplementary Material**. Further inquiries can be directed to the corresponding author.
