# Supplementary material for: A multi-dimensional omics framework identifies GPR35 as a driver of M2 macrophage activation and poor prognosis in colorectal cancer
Source: Front Immunol. 2026 Feb 18;17:1783260. doi: 10.3389/fimmu.2026.1783260 (PMC12957154; doi:10.3389/fimmu.2026.1783260)
Supplement: Supplementary file 1 [file Table1.docx]

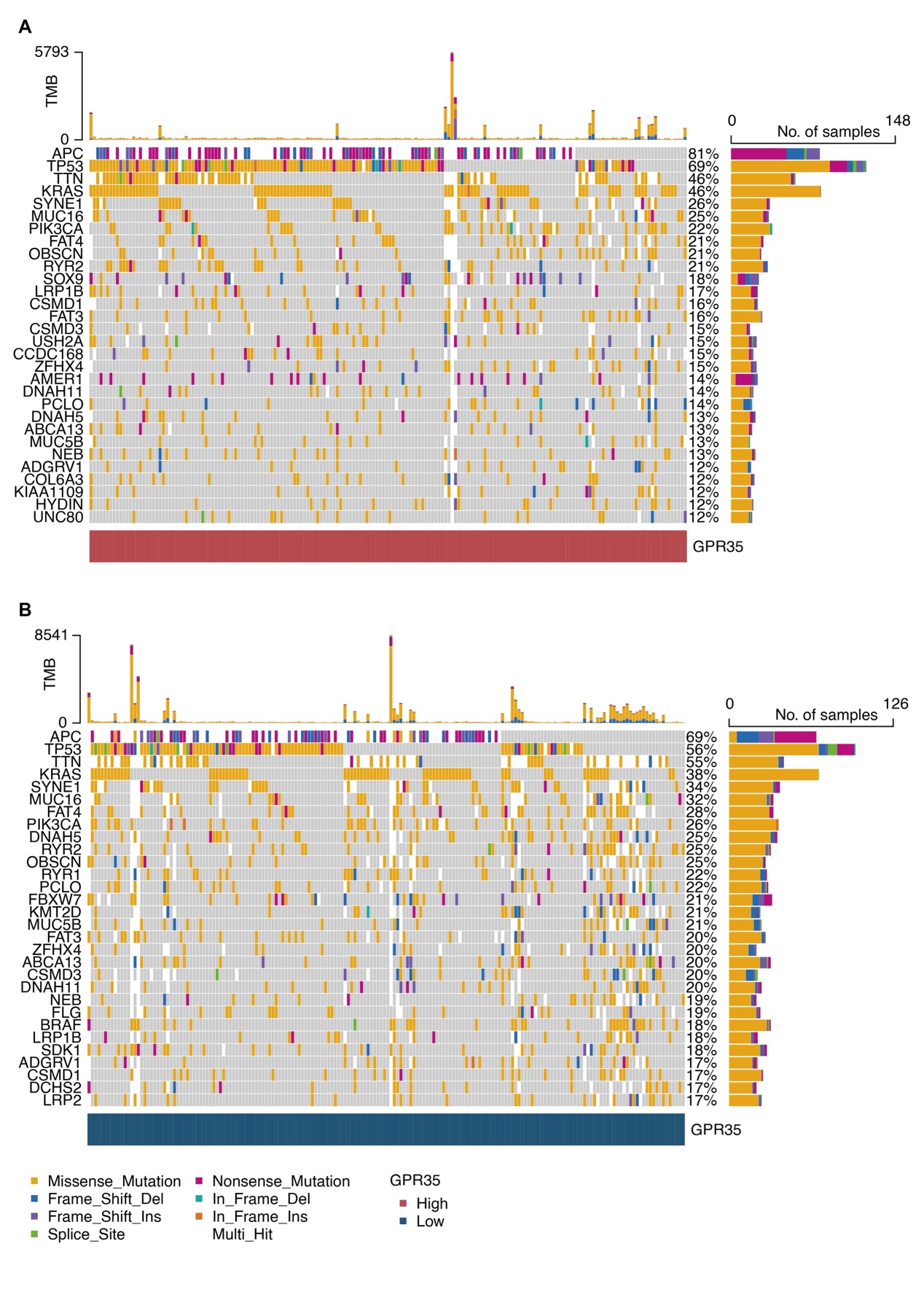


Figure S1. Mutation characteristics related to GPR35. A. Waterfall plot shows the top-ranked mutated genes in the high GPR35 group. B. Waterfall plot shows the top-ranked mutated genes in the low GPR35 group.


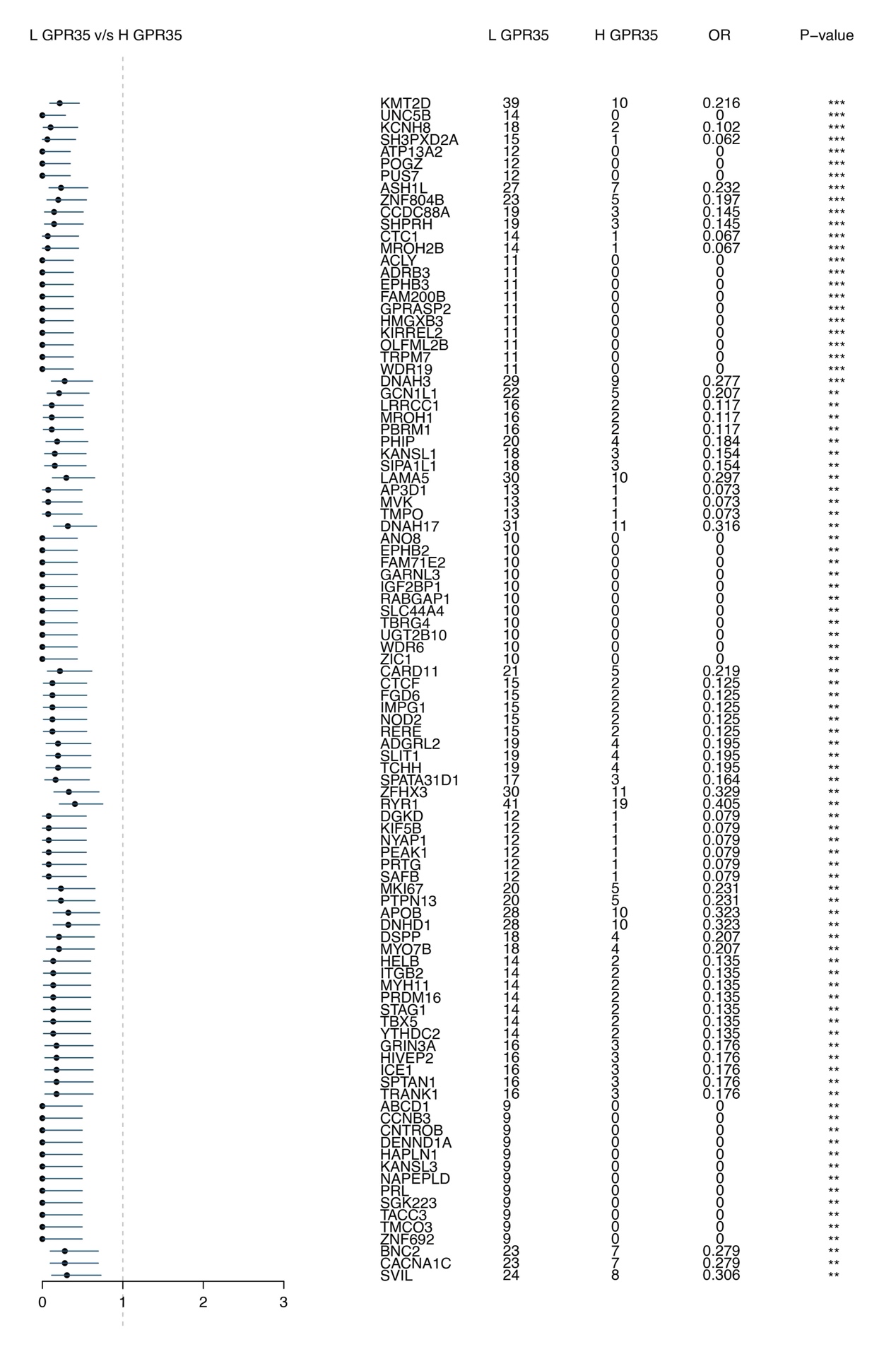


Figure S2. The differentially mutated genes in GPR35-based groups.
